# Management of Abdominal Paraganglioma: A Single Center’s Experience

**DOI:** 10.3390/medicina60040604

**Published:** 2024-04-06

**Authors:** Enrico Battistella, Luca Pomba, Marica Mirabella, Riccardo Toniato, Giuseppe Opocher, Antonio Toniato

**Affiliations:** 1 Endocrine Surgery Unit, Department of Surgery, Veneto Institute of Oncology, IOV-IRCCS, Via Gattamelata 64, 35128 Padua, Italy; 2 School of Medicine, University of Padua, Via Giustiniani 2, 35128 Padua, Italy; 3 Veneto Institute of Oncology, IOV-IRCCS, Via Gattamelata 64, 35128 Padua, Italy

**Keywords:** paragangliomas, pheochromocytoma, neuroendocrine tumors, endocrine surgery

## Abstract

*Background and Objectives:* Paragangliomas (PGLs) are rare neuroendocrine extra-adrenal tumors that could be secreting mass. The symptoms are the typical triad of paroxysmal headache, hypertension and sweating, but could also be accompanied by symptoms involving multiple organs. Surgery is the gold standard treatment for both PGLs and pheochromocytomas (PHEOs). *Material and Methods:* We used a computerized endocrine surgery registry to record the demographic and clinical data of 153 patients who underwent surgery for PPGL between 2010 and 2023 at our hospital. *Results:* Thirteen patients (8.43%) with paragangliomas underwent surgery at our institute. Five patients presented symptomatic syndrome. Preoperative investigations included enhanced abdominal CT (nine patients) and enhanced MRI (seven patients). In cases of suspicious mass, we performed 131I-MIBG scans (two patients) or 68GA-DOTATOC PET-CT scans (11 patients). Laparoscopic approach was used in four cases (30.7%) and abdominal laparotomy in the other nine (69.3%). Biochemical tests were performed on all patients. *Conclusions:* In this retrospective study, we discuss the multidisciplinary management in our institute of this rare disease, from its challenging diagnosis to the surgical strategy for PGLs. Laparoscopic surgery is the gold standard, but a tailored approach should be adopted for each patient.

## 1. Introduction

Paragangliomas (PGLs) are neuroendocrine extra-adrenal tumors originating from chromaffin cells located in the region of autonomic nervous system ganglia. Traditionally, no distinction is made between PGLs and pheochromocytomas (PHEOs). Indeed, as reported in the 2022 WHO classification, “Pheochromocytoma is a neuroendocrine neoplasm that originates from chromaffin cells of the adrenal medulla and is an intra-adrenal paraganglioma” [1]. PGLs are divided into two groups based on their clinical and biological behavior: parasympathetic and sympathetic. Sympathetic PGLs and PHEOs frequently secrete catecholamines, such as noradrenaline and/or adrenaline, predisposing patients to cardiovascular disease, gastrointestinal complications and other endocrine problems, while paragangliomas are usually non-secreting (>95%) [2]. PGLs and PHEOs (jointly, PPGLs) occur in about 2–8 per million people per year [3]. Past literature suggesting that 10% of PPGLs were familial has been superseded by evidence that more than 40% of patients with any PG carry germline mutations [4]. The most important genes that have already been associated with PPGL are NF1, RET, SDHA, SDHB, SDHC, SDHD, TP53 and VHL [4]. PPGLs occur in 10% to 30% of patients with VHL. VHL syndrome is classified as type 1, in which PPGL does not manifest, or type 2, which is subdivided into three subtypes: 2A (encompassing PPGL plus retinal and CNS hemangioblastomas, with low risk for renal carcinoma), 2B (PPGL plus retinal and CNS hemangioblastomas and kidney and pancreatic tumors) and 2C (PPGL only) [5].

The diagnosis of PG remains a clinical challenge. Symptoms of functional PGL are the same as those of PHEO and are related to the excess catecholamine; they include headache, hypertension, pallor, palpitations, sweating, diaphoresis and anxiety. However, most PGLs are diagnosed incidentally or in a screening program for hereditary PGL. Diagnosis requires biochemical tests by measuring blood and urine metanephrines. The current gold standard is a plasma metanephrine (MN) measurement that achieves a sensitivity of 99% for sporadic and hereditary functioning PPGL and a specificity of 99% for hereditary PPGL (and 89% for sporadic), superior to any combination of tests. Normal plasma MN virtually excludes functioning PPGL. Imaging is used, including CT or MRI, in order to acquire a detailed description of the PG and its boundaries, especially in the aorta and cava vein, and functional imaging with 68GA-DOTATOC PET-CT or 131I-metaiodobenzylguanidine (131I-MIBG) scans is also conducted to identify plurifocal lesions [6]. Enhanced CT has a higher sensitivity and can locate PPGLs as small as around 5 mm; in 100% of cases, PPGLs show a mean attenuation of more 10 HU [7].

The mainstay of PPGL treatment is surgical removal. Surgical manipulation of the tumor leads to massive release of catecholamines (catecholamine storm) and has the potential to cause hypertensive crisis, cardiac arrhythmias, myocardial ischemia, pulmonary edema and stroke [8]. Current guidelines and retrospective studies support the initiation of preoperative antihypertensive therapy with selective (doxazosin, prazosin, terazosin) or nonselective (phenoxybenzamine) alpha adrenergic blockers in patients with symptoms of catecholamine excess [9]. Management of malignant PPGLs includes a combination of surgical debulking, medical management to control symptoms of catecholamine excess (metyrosine), radionuclide therapy (131I-MIBG or somatostatin analogues), chemotherapy (cyclophosphamide, vincristine, dacarbazine combination) and external beam radiation therapy [10]. In the 2017 WHO classification of endocrine tumors, all PPGLs were considered malignant neoplasms with varying metastatic potential, in the same way as epithelial neuroendocrine neoplasms, and this was maintained in the 2022 WHO classification [1,11]. The current TNM of staging patients with PPGL is challenging because it is impossible to differentiate between benign and malignant tumors. Nevertheless, TNM staging may help in the follow-up and treatment of patients with PPGL; therefore, this staging system should be based on the recognition of clinical predictors of metastases and survival in the context of metastatic disease [1,12]. In this retrospective study, we present our management of the disease from a surgical point of view, and we discuss the current evidence and recommendations for managing this potentially life-threatening condition.

## 2. Materials and Methods

In this retrospective, single-center study, we used a computerized endocrine surgery registry to record the demographic and clinical data of 153 patients who had surgery for PPGLs between 2010 and 2023 in our hospital, which is a tertiary referral clinic for endocrine surgery. We only included patients operated for abdominal PGLs, excluding patients under 18 years old and patients that underwent surgery for PHEOs. The clinical characteristics of these patients included sex, age, pre-existing PPGL symptoms, mutation status and family history. The patients underwent biochemical screening (urinary and plasmatic catecholamines or metanephrines) and radiological imaging (an initial computed tomography scan (CT) or magnetic resonance imaging (MRI) of the abdomen and pelvis). They also underwent functional imaging for incidental lesions highly suspicious for PPGL with inconclusive biochemical testing. Postoperative results and operative complications were also recorded.

Each patient was discussed in a multidisciplinary meeting to create a comprehensive and tailored treatment plan from diagnosis to surgical approach.

The surgical intervention was performed by the same endocrine team at the Endocrine Surgery Unit in the Castelfranco Veneto Hospital. The surgery was chosen as either open surgery or transperitoneal lateral approach, depending on the dimensions of the lesion (all lesions >4 cm were treated with open surgery) and position of the tumor. The anterior transperitoneal approach was always performed in the same way and involved the patient being placed in a supine decubitus position. Four trocars were used (the optical trocar was placed just above the umbilicus).

The patients continue to follow-up every year at the clinic.

Written informed consent was obtained from each participant following full explanation of the study.

## 3. Results

In the study period, 153 patients underwent surgery for PPGLs, of which 13 patients (8.43%) had paragangliomas. All patients originated from northern Italy and included eight females (61.5%) and five males (38.5%) (F:M = 1.6:1). The median age at presentation of the PGL was 47.2 years (range 30 to 69 years). The PGL diagnoses were as follows: five patients presented PPGL-related symptoms including hypertension resistant to medical treatment, diaphoresis, migraine and palpitations; two patients were diagnosed during diagnostic imaging for unspecific abdominal pain; four patients were diagnosed during screening for genetic syndromes (two patients affected by Von Hippel Lindau syndrome and another two patients with SDH germline mutation); and the last two patients were diagnosed with PGL during follow-up for neoplastic disease (breast tumor and Hodgkin’s lymphoma).

Two patients had undergone previous surgery in anamnesis: two patients had undergone bilateral adrenalectomy, one patient for bilateral PHEO in VHL syndrome and one patient who also performed a total thyroidectomy for MEN2B syndrome. All patients underwent 24 h urine tests for metanephrine and catecholamines as well as plasma metanephrine and catecholamine tests. The tests results were above the normal range in eight patients. Other hormonal tests, including for renin and aldosterone, did not show abnormalities of clinical significance.

Preoperative investigations, including enhanced abdominal CT (nine patients) and enhanced MRI (six patients) were performed in order to localize the PGL successfully. In cases of a suspicious mass, 131I-MIBG scans (2 patients) or 68GA-DOTATOC PET-CT scans (11 patients) were used as another important diagnostic tool to confirm the PGL.

Tumor size varied between 2.5 cm and 6 cm.

Preoperative antihypertensive therapy was performed for patients with sustained or paroxysmal hypertension as well as normotensive patients. An alpha-adrenergic blocker was administered starting at least three weeks preoperatively using doxazosin from 2 to 16 mg/day to optimize blood pressure and ensure euvolemia for volume expansion. The addition of a β-adrenergic antagonist to the preoperative regimen was determined by the extent of catecholamine-induced tachycardia. Treatment with a β-adrenergic antagonist was never initiated before adequate alpha blockade.

**Figure 1:**
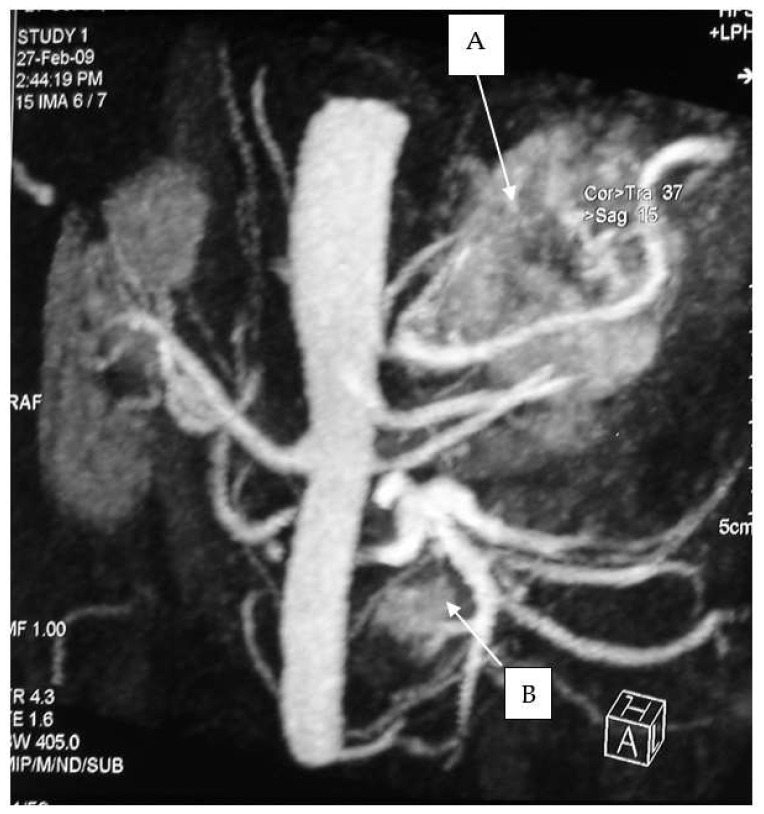
Adrenal pheochromocytoma (A) and abdominal paraganglioma under the left renal vein (B).

**Figure 2:**
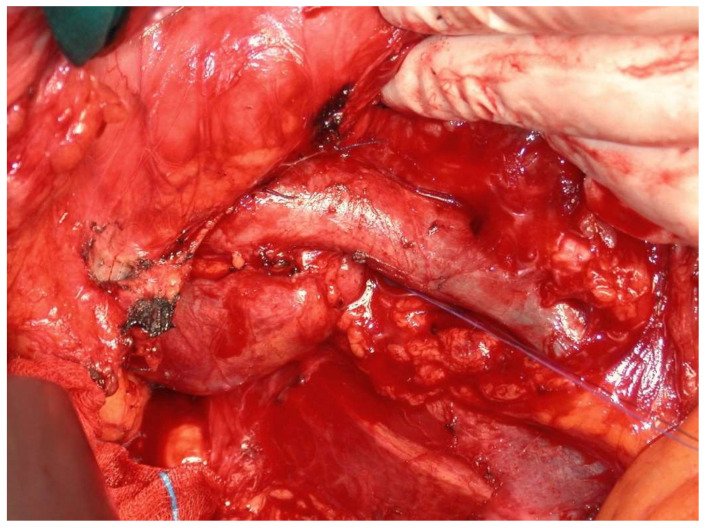
Malignant paraganglioma with peritoneal carcinosis.

All operations were performed by the same endocrine surgical team. The surgical approaches chosen were laparoscopic excision with transperitoneal approach and abdominal laparotomy based on the results of preoperative investigations considering the position of the mass and the bleeding risk. Laparoscopic approach was performed in four cases (30.7%) and abdominal laparotomy in the other nine (69.3%). Combined abdominal and neck surgery was performed in one case of dual-localization PGL: peri-carotidal and para-aortic. In addition, we performed one left adrenalectomy for the excision of a PGL under the left renal vein using surgical laparotomy, and in another case, cytoreductive surgery was carried out due to the intraoperative discovery of carcinosis (Figure 1 and Figure 2). This patient died eight years later, with massive thorax and abdominal metastases. Another patient died three years after the surgical intervention for malignant hypertension due to metastatic PHEO. The follow-ups for the other 11 patients showed them to be free from disease following operation (follow-up time from 1 year to 10 years).

The results of the study are summarized in Table S1.

## 4. Discussion

PHEOs and sympathetic PGLs are neuroendocrine-related tumors with clinical symptoms caused by the excessive secretion of catecholamines. The typical triad of PPGL symptoms is paroxysmal headache, palpitations and heavy sweating. Other symptoms are hypertension, chest pain, psychiatric symptoms, hyperglycemia, constipation and syncope. Less common symptoms include Takotsubo syndrome, other life-threatening cardiovascular events and preeclampsia [12]. A non-responsive hypertension to medical treatment was recorded in five patients (38%) included in the study. Patients also complained of migraines, palpitations and headaches.

PGLs represent less than 20% of all PPGLs, limiting considerations of technical surgery. The incidence of PPGL is low and estimated to be 2–8 cases/million people; furthermore, malignant PPGLs account for 14–17% of the total, and this means fewer than 1 per million people per year [3,4]. In this study, we reported the general characteristics of 13 patients with abdominal paragangliomas. The female-to-male ratio was 1.6; female predominance has also been reported in some previous studies [11]. However, in a retrospective study on 10 patients, Darouassi et al. reported a male predominance with a sex ratio of 2.33 [12]. PGLs are commonly diagnosed between the ages of 40 and 70, although these neoplasms can occur over a wide age range. They appear at a younger age when they occur as part of a hereditary syndrome. In our study, the average age of diagnosis was 47.2 years, while if we consider only patients with genetic mutation, the average age is 38.4 years, so these data are in accordance with the literature.

PG is predominantly of interest to molecular biologists, endocrinologists and nuclear radiologists who research new genetic mutations, markers and imaging modalities that can guarantee an early diagnosis, in addition to new modern therapies based on metabolism and modifications of neuroendocrine cells. For this reason, multidisciplinary treatment has become routine for PGL because of the complexity of diagnosis, treatment and post-operative care.

As reported int the Results section, five patients presented genetic mutations (38.4%). Indeed, four PGLs were diagnosed during screening for genetic syndromes (two patients affected by Von Hippel Lindau syndrome and another two patients with SDH germline mutation), and one patient had a known MEN2B-mutation; this patient was a young woman (32 years old) who had already undergone a total thyroidectomy for medullary thyroid carcinoma before abdominal surgery. One patient affected by VHL had undergone a bilateral adrenalectomy for bilateral PHEO, and the other patient affected by VHL reported a detachment of the retina due to retinal angiomatosis.

In the literature, up to 30% of PPGL patients report a positive family history with an identifiable hereditary syndrome, including Von Hippel Lindau disease (VHL), multiple endocrine neoplasia type 2 (MEN 2), type 1 neurofibromatosis (NF1) and familial PPGL syndrome that occur secondary to SDHA/B/C/D germline gene mutations [13,14].

The best biochemical method for diagnosing PGL is free-plasma and urinary metanephrine and normetanephrine testing, free-plasma dopamine testing and chromogranin A testing. Another catecholamine metabolite, methoxy tyramine, measured in plasma, has been shown to be an important biomarker for metastatic PGL. Guidelines accept both plasma-free and urinary MNs for the screening of PCCs and PGLs, with both determinations being considered to have similar sensitivities (97%) and specificities (91%) [15,16].

All the patients in the study with suspected PGL underwent 24 h urine tests for metanephrine and catecholamines, as well as plasma metanephrine and catecholamine tests, with above-normal results found in eight patients (61.5%).

**Figure 3:**
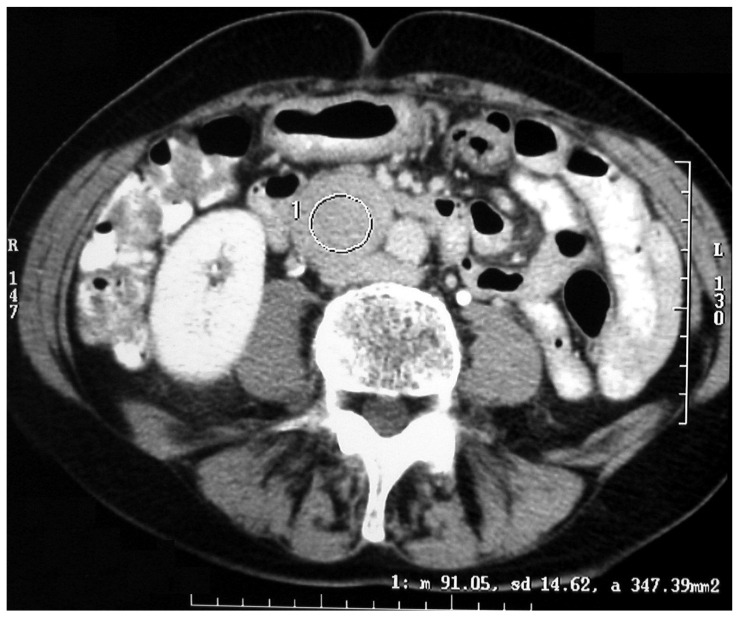
CT scan of an abdominal paraganglioma of above the aortic bifurcation (Zuckerkandl organ’s).

**Figure 4:**
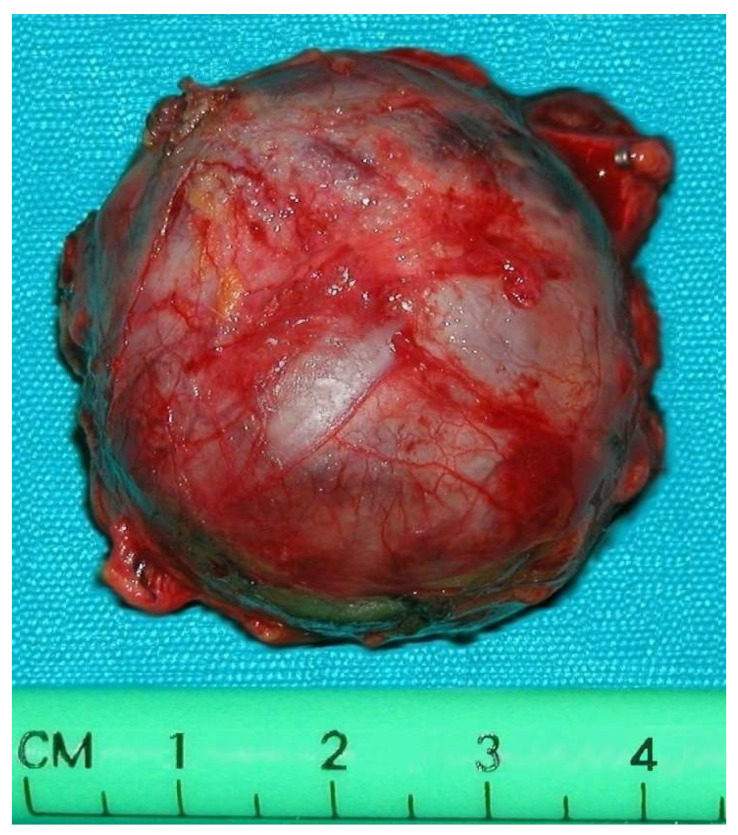
Pathological specimen of the abdominal paraganglioma reported in Figure 3.

The diagnostic localization of a PCC/PGL is traditionally attempted only once excess catecholamine has been biochemically ascertained [17,18]. Both computed tomography (CT) scanning and MRI are mainstays for adrenal imaging and are used as the primary tests for localizing catecholamine-secreting tumors. CT is the preferred method for its excellent resolution (Figure 3 and Figure 4).

If CT scans and/or MRI fail to detect the tumor, or if there is concern that the patient has a metastatic disease owing to a large primary tumor size, functional localization can be attempted using PET scanning with several radiolabeled ligands (DOPA, 18F-FDG and 18F-FDA), 123I-metaiodobenzylguanidine (MIBG) scintigraphy and somatostatin receptor imaging (68-Ga DOTATOC). Several clinical researchers have reported that lesion detection sensitivity with 68-Ga DOTATOC is higher than with other conventional functional imaging techniques, especially in metastatic and multifocal primary diseases. Its use is recommended in parallel with ^18^FDG PET-CT [19,20].

Surgical excision remains the mainstay of treating PGL, especially because it is currently impossible to differentiate benign from malignant tumors. Pre-operative management using selective alpha-adrenergic blockers, such as doxazosin, prazosin and calcium channel blockers, has been applied to minimize the risk of perioperative hemodynamic instability in patients with functional PPGL [21]. The most important part of managing these patients is always maintaining communication between the surgeon and anesthesiologist throughout the pre-operative, peri-operative, and postoperative stages [22,23]. In our series, the pre-operative medicament was the same in PHEO and PGL, considering them the same entity in different positions [24].

Only experienced surgeons and anesthesiologists should be assigned to PPGL surgeries. From a surgical point of view, laparoscopic surgery is the preferred approach for PGL tumor resection, almost as a starting point thanks to the considerable knowledge on laparoscopic adrenalectomy. The laparoscopic approach should not be chosen for cases of suspected metastases and tumor sizes greater than 6 cm, which are conditions under which traditional open access surgery is mandatory. Even with expert surgical skills, tumors located between the inferior vena cava and aorta—pericaval and para-aortic—especially beginning from the aortic bifurcation and extending to the diaphragmatic crura, remain challenging from a surgical point of view and most such cases exclude laparoscopy due to the very long mean operating time (55–600 min). PGLs located at the level of the pancreas or diaphragmatic crura can prove very difficult to operate on, even in open surgery [25]. For these reasons, we chose a laparoscopic approach in only four cases. In our case series, there was close adherence of the PGLs to the great vessels, and the safest way to excise the tumors with intact capsules was the open abdomen approach.

The anterior trans-peritoneal laparoscopic approach is the gold standard technique, while the retroperitoneal approach can be used for small-size PGs located above the renal veins. At our clinic, we use the anterior trans-peritoneal approach due to the simpler anatomic references. The transperitoneal laparoscopic approach has been widely used for adrenal tumors of all sizes due to the excellent anatomical exposure. The patient is placed in the lateral decubitus position, and the table is flexed to maximize the space between the costal margin and the iliac crest [25,26,27].

The conversion from laparoscopy to laparotomy can be necessary due to adhesions between the PGL and blood vessels and consequent risk of intra-operative bleeding. PGLs usually have short blood vessels and bleeding can only be stopped by underpinning the wall of the aorta or inferior cava vein, which is extremely difficult during laparoscopy. This is also why laparoscopy’s operating time cannot be shortened and many experienced surgeons prefer laparotomy in this situation. It also allows better control of blood vessels and greater radicality in the case of suspected malignancies or treatment of metastases. The incidence of deep vein thrombosis in laparoscopic adrenalectomy can reach up to 4% of patients; therefore, antithrombotic prophylaxis should be considered according to clinical thromboprophylaxis guidelines [27].

Surgical resection or debulking of primary tumors, although rarely curative even with the complete resection of metastases, is appropriate in select patients with metastatic PPGLs and may prevent local complications and compression and improve quality of life, lowering rates of hormonal morbidity and potentially improving survival [28,29]. In one case, we found peritoneal carcinosis with a peritoneal cancer index of 12 points, involving the pelvic peritoneum and the mesentery of small bowel. A complete cytoreductive surgery was performed, and the patient was free from disease for almost seven years, and after 8 years, he died. Targeted radionuclide therapies (TRTs), immunotherapy and tyrosine kinase inhibitors can also be used either alone or in combination as multimodality therapy [30,31]. Our study has several limitations: first, the retrospective design did not permit us to obtain complete information about genetic mutation; second, the small number of the enrolled patients is limiting, although the rarity of chromaffin tumors should be considered.

## 5. Conclusions

This extremely rare disease should be managed in a referral center where a multidisciplinary team can develop a personalized treatment for each patient. From a surgical point of view, the laparoscopic approach is the gold standard, but surgeon experience and the dimensions and position of the PGLs must be considered to guarantee the safety of patients preferring open access.

## Data Availability

Data were obtained from the patients’ medical records.

## Supplementary Materials

The following are available online at www.mdpi.com/xxx/s1, Table S1: Clinical features and management of each patient included in the study.
