# Supplementary material for: Management of Abdominal Paraganglioma: A Single Center’s Experience
Source: Medicina (Kaunas). 2024 Apr 6;60(4):604. doi: 10.3390/medicina60040604 (PMC11051844; doi:10.3390/medicina60040604)
Supplement: Supplementary file 1 [file medicina-60-00604-s001.zip › medicina-2870192-supplementary.pdf]

	Surgery Age	Sex	Clinical Presentation	Genetic Mutation	Metanephrine and cathecolamines plasmatic and urinary	Imaging	Mass Size and localization	Surgery	FU
**Patient n°1**	32 yrs	F	Hypertension and migraine in MEN2B	RET	POSITIVE	Enhanced CT DOTATOC	20 x 30 mm inter-aortocaval PGL	VLS mass excision	Died after 3 years for metastases
**Patient n°2**	42 yrs	M	Hypertension resistent to medicament	/	POSITIVE	Enhanced CT DOTATOC	30 mm + 30 mm bilateral peri-caval PGL under the renal vein	Explorative laparotomy: cytorecductive surgery	Died after 8 years for metastases
**Patient n°3**	56 yrs	M	Abdominal Pain	/	NEGATIVE	US Enhanced CT MIBG	40x35x38 mm Zuckerkandl organ	VLS mass excision	Negative after 10 yrs
**Patient n°4**	49 yrs	M	Hypertension	/	POSITIVE	Enhanced CT DOTATOC	20 x 20 left PHEO and 25x25 mm right retroperitoneal PGL	PGL Laparotomic excision and left adrenalectomy	Negative after 10 yrs
**Patient n°5**	35 yrs	F	Familial screening	SDHD mutation	NEGATIVE	Enhanced CT Enhanced MRI DOTATOC	30 mm abdominal PGL+ 20 mm neck PGL	VLS mass excision and cervicotomy	Negative after 10 yrs
**Patient n°6**	44 yrs	M	VHL-syndrome	/	POSITIVE	Enhanced MRI DOTATOC	27x23 mm para-aortic PGL	VLS mass excision	Negative after 1 yrs
**Patient n°7**	56 yrs	F	Cancer FU	/	POSITIVE	Enhanced CT Enhanced MRI DOTATOC	60 x 30 mm para-aortic PGL	Laparotomic excision	Negative after 1 yrs
**Patient n°8**	30 yrs	F	VHL-syndrome	RET	POSITIVE	Enhanced CT Enhanced MRI DOTATOC	50 x30 mm Peri-caval under left renal vein	Laparotomic excision	Negative after 5 yrs

**Patient n°9**	69 yrs	F	Hypertension and headache	/	POSITIVE	Enhanced CT<br>Enhanced MRI<br>DOTATOC	45 mm<br>Left Para-aortic abdominal PGL	Laparotomic excision	Negative after 5 yrs
**Patient n°10**	53 yrs	F	Abdominal Pain	/	NEGATIVE	Enhanced MRI<br>MIBG	60 x 20 mm<br>Peri-caval PGL	Laparotomic excision	Negative after 3 yrs
**Patient n°11**	51 yrs	M	Familial screening	SDHD mutation	POSITIVE	Enhanced MRI<br>DOTATOC	42x36x42 mm<br>Peri-caval above the right renal vein PGL	Laparotomic excision	Negative after 1 yrs
**Patient n°12**	46 yrs	F	Cancer FU	/	NEGATIVE	Enhanced CT<br>DOTATOC	50 mm retroperitoneal peri-adrenal PGL	Lombotomic Excision	Negative after 1 yrs
**Patient n°13**	39 yrs	F	Abdominal pain	/	NEGATIVE	US<br>Enhanced MRI<br>DOTATOC	48 x 23<br>Zuckerkandl organ	Laparotomic excision	Negative after 8 years

Table S1: Clinical features and management of each patient included in the study.
